# Transforming growth factor β (TGFβ) induces NUAK kinase expression to fine-tune its signaling output

**DOI:** 10.1074/jbc.RA118.004984

**Published:** 2019-01-08

**Authors:** Constantinos Kolliopoulos, Erna Raja, Masoud Razmara, Paraskevi Heldin, Carl-Henrik Heldin, Aristidis Moustakas, Lars P. van der Heide

**Affiliations:** From the ‡ Department of Medical Biochemistry and Microbiology, Science for Life Laboratory, Box 582 Biomedical Center, Uppsala University, 751 23 Uppsala, Sweden and; the § Ludwig Institute for Cancer Research, Science for Life Laboratory, Box 595 Biomedical Center, Uppsala University, 751 24 Uppsala, Sweden

**Keywords:** AMP-activated kinase (AMPK), cell cycle, epithelial-mesenchymal transition (EMT), myofibroblast, SMAD transcription factor, signal transduction, transforming growth factor beta (TGF-beta), cell signaling, NUAK family kinase

## Abstract

TGFβ signaling via SMAD proteins and protein kinase pathways up- or down-regulates the expression of many genes and thus affects physiological processes, such as differentiation, migration, cell cycle arrest, and apoptosis, during developmental or adult tissue homeostasis. We here report that NUAK family kinase 1 (*NUAK1*) and *NUAK2* are two TGFβ target genes. NUAK1/2 belong to the AMP-activated protein kinase (AMPK) family, whose members control central and protein metabolism, polarity, and overall cellular homeostasis. We found that TGFβ-mediated transcriptional induction of *NUAK1* and *NUAK2* requires SMAD family members 2, 3, and 4 (SMAD2/3/4) and mitogen-activated protein kinase (MAPK) activities, which provided immediate and early signals for the transient expression of these two kinases. Genomic mapping identified an enhancer element within the first intron of the *NUAK2* gene that can recruit SMAD proteins, which, when cloned, could confer induction by TGFβ. Furthermore, NUAK2 formed protein complexes with SMAD3 and the TGFβ type I receptor. Functionally, NUAK1 suppressed and NUAK2 induced TGFβ signaling. This was evident during TGFβ-induced epithelial cytostasis, mesenchymal differentiation, and myofibroblast contractility, in which NUAK1 or NUAK2 silencing enhanced or inhibited these responses, respectively. In conclusion, we have identified a bifurcating loop during TGFβ signaling, whereby transcriptional induction of NUAK1 serves as a negative checkpoint and NUAK2 induction positively contributes to signaling and terminal differentiation responses to TGFβ activity.

## Introduction

A dynamic balance of TGFβ^6^ family signaling pathways determines whether cells undergo differentiation, arrest of proliferation, migration, or apoptosis, which altogether shape the direction of embryogenesis and maintain tissue homeostasis (1–4). TGFβ signaling initiates when the ligand binds to its type II receptor (TβRII), which recruits and phosphorylates the type I receptor (TβRI) (5, 6). Activated TβRI binds and phosphorylates receptor-activated SMADs (R-SMADs) (*i.e.* SMAD2 and SMAD3), which further interact with a common SMAD (co-SMAD), SMAD4. Upon accumulation in the nucleus, SMAD complexes together with transcription factors regulate gene expression (2, 6). TGFβ receptors also recruit ubiquitin ligases and protein kinases, leading to activation of the mitogen-activated protein kinase (MAPK) family members, p38, c-Jun N-terminal kinase, and ERK1/2 (2). The MAPK signals, coordinately with SMADs, mediate the physiological responses to TGFβ.

Earlier microarray screening in human breast cancer cells yielded salt-inducible kinase (SIK) as a gene that is transcriptionally induced in response to TGFβ signaling (7, 8). SIK functions together with the inhibitory SMAD (I-SMAD) SMAD7 and the ubiquitin ligase Smurf2 to negatively regulate TGFβ receptor signaling by promoting TβRI turnover (7, 9). SIK is one of 14 serine/threonine kinases of the AMP-activated protein kinase (AMPK) family, which regulate metabolism, cell cycle, and polarity (10). The liver kinase B1 (LKB1), a tumor suppressor kinase, the TGFβ-activated kinase 1 that is activated by the TGFβ receptor complex via ubiquitination, and the calcium/calmodulin protein kinase kinase β can phosphorylate and activate the AMPKs (11). Some AMPKs are transiently transcriptionally induced, whereas others can be regulated by allosteric cofactors, such as AMP, or by ubiquitination (12). The prototype AMPKs phosphorylate the tuberous sclerosis complex 2 protein and inhibit the mammalian target of rapamycin (mTOR) complex 1 kinase, suppressing mRNA translation and cell proliferation (10, 13).

Influenced from the evidence on *SIK* acting downstream of TGFβ signaling (7–9), we performed a screen of all AMPKs expressed in two TGFβ-responsive cell models, a mouse mammary epithelial cell and a human skin fibroblast, and found that *Nuak1/NUAK1* and *Nuak2/NUAK2* mRNAs are induced in response to TGFβ. The novel (nua) kinase (NUAK) subfamily consists of two members, NUAK1 or AMPK-related kinase 5 (ARK5) and NUAK2 or sucrose nonfermenting AMPK-related kinase (SNARK). *NUAK2* can be transcriptionally induced by UV light (14) and is activated under DNA damage; oxidative, glucose, or glutamine deprivation stress; and high AMP or low ATP levels (15). NUAK2 can be induced during muscle differentiation, protecting myocytes from undergoing apoptosis (16). NUAK2 regulates the myosin regulatory light chain (MLC) phosphatase via myosin-phosphatase Rho-interacting protein (17). NUAK2 phosphorylates and inhibits MYPT1, the regulatory subunit of MLC phosphatase, stabilizing actin filaments and mediating contraction of smooth muscle cells (17).

Pathologically, NUAK2 regulates hepatitis C virus replication and enhances TGFβ signaling and hepatic fibrosis (18). In melanomas, NUAK2 affects cell cycle progression and migration (19, 20), whereas it affects gene expression in human cervical cancer cells under stress (21). Tumor necrosis factor α and CD95 induce NUAK2 expression in breast cancer cells to promote invasiveness and survival (22).

NUAK1 physically interacts with MYPT1 and phosphorylates and inhibits its phosphatase activity, enhancing phosphorylation of MLC2 (23). NUAK1 contains a predicted AKT phosphorylation motif, which, when phosphorylated, results in elevated phosphorylation of the ataxia-telangiectasia protein and of p53, promoting survival (24). Accordingly, NUAK1 suppresses apoptosis induced by nutrient starvation and death receptors in hepatoma cells (24). NUAK1 can also modulate AMPK activity and therefore ATP levels in Myc-driven tumors, by limiting mTOR signaling. NUAK1 depletion released pro-apoptotic signals both *in vitro* and *in vivo* in hepatocellular carcinoma (25), establishing NUAK1 as a survival factor for tumor cells. Furthermore, NUAK1 can activate the polo kinase-1 indirectly, via inhibition of protein phosphatase 1β, thus stimulating cell cycle progression through the S phase (26). Moreover, elevated NUAK1 levels can drive invasion of pancreatic cancer or exert tumor-promoting effects in breast cancer (27, 28). On the other hand, NUAK1 can be anti-tumorigenic, by binding and phosphorylating p53 in a LKB1 activation–dependent manner, by inducing expression of the cell cycle inhibitor p21 and G_1_/S phase arrest (29). In normal diploid fibroblasts, NUAK1 is induced upon aging, mediating senescence (30), further supporting a tumor-suppressing function.

The present study ascribes novel functions to NUAK1 and NUAK2. Transcriptional induction of NUAK1 and NUAK2 by TGFβ generates signaling loops in a way that NUAK1 inhibits, whereas NUAK2 promotes, biological responses mediated by TGFβ signaling.

## Results

### TGFβ transcriptionally induces Nuak1/NUAK1 and Nuak2/NUAK2 in a SMAD- and kinase-dependent manner

**Figure 1.**
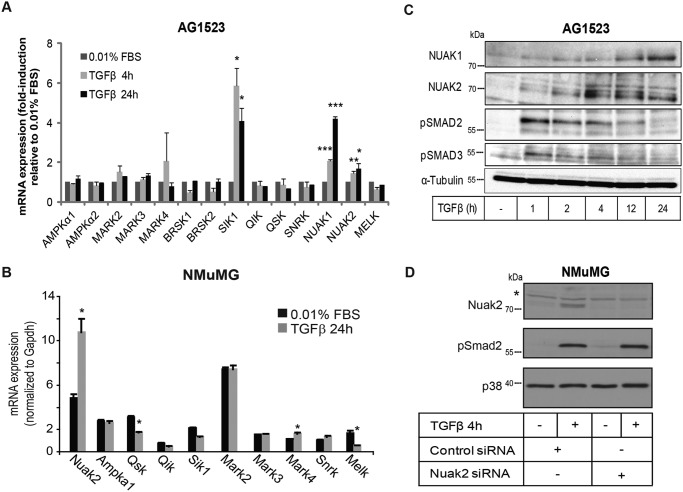
**TGFβ induces NUAK1 and NUAK2 expression in primary fibroblasts and epithelial cells.** *A*, relative mRNA expression of AMPKs and AMPK-related kinases normalized to basal levels expressed in primary fibroblasts AG1523 as measured by real-time qRT-PCR, with and without TGFβ (1 ng/ml) stimulation for 4 and 24 h. *B*, mRNA expression of AMPKs and AMPK-related kinases expressed in NMuMG, as measured by real-time qRT-PCR, with and without TGFβ (5 ng/ml) stimulation for 24 h and normalized to *Gapdh. C*, immunoblot analysis of NUAK1 and NUAK2 in AG1523 cells at the indicated time periods of TGFβ (1 ng/ml) stimulation. Phospho-SMAD2 and phospho-SMAD3 serve as positive controls for TGFβ activity. α-Tubulin serves as a protein-loading control. Molecular size markers in kDa are shown. *D*, imunoblotting for NUAK2, phospho-SMAD2, and total p38 levels in NMuMG cells after adding fresh medium containing TGFβ (5 ng/ml) for 1 h. Total p38 MAPK serves as a protein-loading control. A *star* shows nonspecific protein bands. Molecular size markers in kDa are shown. All bar graphs show average values derived from triplicate determinations and the corresponding S.D. values. Graphs show mean ± S.E. (*error bars*) from at least three independent experiments. *, *p* < 0.05; **, *p* < 0.01; ***, *p* < 0.001; statistically significant compared with the non-TGFβ–treated samples.

By screening for expression of 15 AMPK members and related kinases in human foreskin AG1523 fibroblasts and in mouse mammary epithelial NMuMG cells that respond well to TGFβ, we found that many AMPKs were expressed in both cell types, whereas some kinases were essentially undetectable (Fig. 1 (*A* and *B*); data not shown). We asked whether TGFβ could induce expression of these kinases and found that *NUAK1*, *NUAK2*, and *SIK1* were reproducibly induced by 2-fold or more in the fibroblasts (Fig. 1*A*); *Nuak2* mRNA was induced by about 2-fold in mammary cells (Fig. 1*B*). TGFβ-dependent inducibility was reproduced in diverse cell types, including mouse C2C12 myoblasts and LKB1 knockout MEFs (data not shown), immortalized human mammary epithelial MCF10A cells and their Ras-transformed derivatives (MCF10A-Ras MII), immortalized human keratinocytes HaCaT, and human lung adenocarcinoma A549 cells (Fig. S1). The degree of inducibility of *NUAK1* mRNA, however, varied between cell types (Fig. S1*A*). Protein analysis confirmed that NUAK1 and NUAK2 were induced in a time-dependent manner in human fibroblasts (Fig. 1*C*), mammary cells (data shown for NUAK2 only; Fig. 1*D*), and HaCaT and human cervical carcinoma HeLa cells (Fig. S1*B*). Protein specificity was confirmed by predicted electrophoretic mobility, inducibility by TGFβ stimulation, and loss of protein upon transfection of cells with siRNAs (Fig. 1*D*, *Nuak2*).

**Figure 2.**
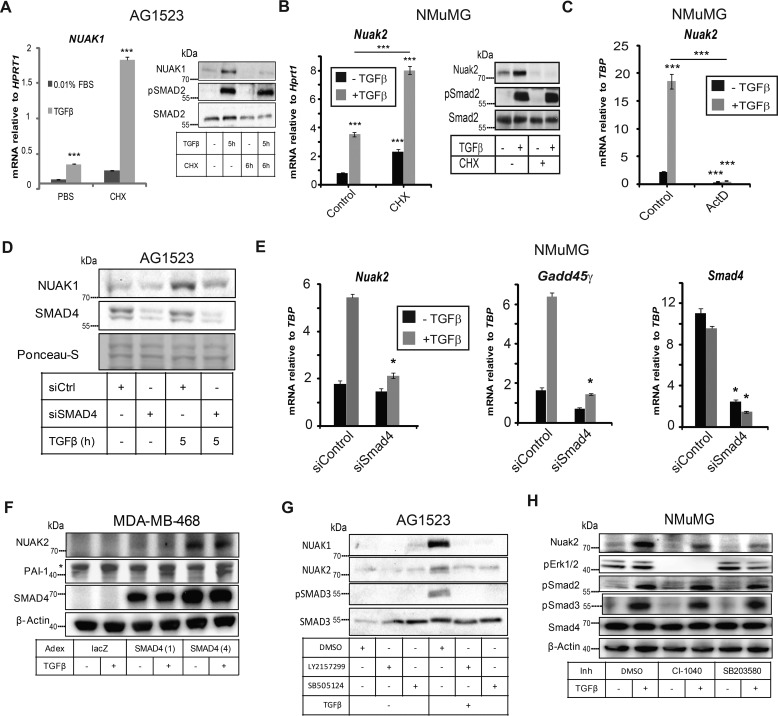
**NUAK1 and NUAK2 are transcriptionally induced by TGFβ.** *A*, real-time qRT-PCR analysis of *NUAK1* mRNA normalized to *HPRT1* mRNA from AG1523 cells after treatment with cycloheximide (20 μm) or an equivalent volume of PBS as negative control for 1 h followed by TGFβ (1 ng/ml) stimulation for 5 h. On the *right*, corresponding immunoblot for NUAK1, phospho-SMAD2 and total SMAD2 under the conditions used in the samples for real-time qRT-PCR. Molecular size markers in kDa are shown. One representative experiment of two is shown. *B*, real-time qRT-PCR analysis of *Nuak2* mRNA normalized to *Gapdh* mRNA from NMuMG cells after treatment with vehicle or cycloheximide for 15 min followed by TGFβ (5 ng/ml) stimulation for 1 h. Immunoblots of NUAK2, phospho-SMAD2, and total SMAD2 proteins serve as controls for the RNA analysis. SMAD2 serves as a protein-loading control. Molecular size markers in kDa are shown. *C*, NMuMG cells were pretreated with vehicle or actinomycin D for 1 h before treatment with TGFβ (5 ng/ml) for 1 h. *Nuak2* mRNA was normalized to *Gapdh* mRNA as measured by real-time qRT-PCR. *D*, immunoblotting of NUAK1 and SMAD4. AG1523 cells were stimulated with TGFβ (1 ng/ml) for 5 h. Ponceau-S staining of the immunoblot serves as a protein-loading control. Molecular size markers in kDa are shown. *E*, mRNA expression of *Nuak2*, *Gadd45*γ, and *Smad4* in NMuMG cells after treatment with control or *Smad4* siRNA and TGFβ (5 ng/ml) stimulation for 1 h. *F*, immunoblots of NUAK2, PAI-1, SMAD4, and β-actin proteins in MDA-MB-468 cells transiently infected with Adex-LacZ or Adex-SMAD4 (the latter at two different multiplicities of infection, 1 and 4) prior to cell starvation and stimulation with TGFβ (5 ng/ml) for 24 h. β-Actin serves as protein-loading control; a *star* shows nonspecific protein bands. Molecular size markers in kDa are shown. *G*, immunoblotting of NUAK1 and NUAK2 in AG1523 cells after treatment with TGFβ (5 ng/ml) for 3 h in the presence of TβRI kinase inhibitors LY2157299 (5 μm) or SB505124 (2.5 μm) or DMSO (0.1%). Molecular size markers in kDa are shown. *H*, immunoblots of NUAK2, phospho-ERK1/2, phospho-SMAD2, phospho-SMAD3, SMAD4, and β-actin proteins in NMuMG cells serum-starved overnight, followed by pretreatment with the indicated inhibitors or vehicle (DMSO) for 1 h prior to stimulation with TGFβ (1 ng/ml) for 6 h. β-Actin serves as protein-loading control. Molecular size markers in kDa are shown. Data are presented as mean ± S.E. (*error bars*) after performing at least three independent experiments. *Asterisks* imply significant differences compared with controls: *, *p* < 0.05; **, *p* < 0.01; ***, *p* < 0.001.

After blocking ribosomal function with cycloheximide, TGFβ induced *NUAK1* mRNA by about 7-fold in AG1523 cells (Fig. 2*A*) and *Nuak2* mRNA by about 4-fold in the NMuMG cells (Fig. 2*B*); these were comparable -fold inductions relative to control without cycloheximide (Fig. 2, *A* and *B*), indicating that TGFβ regulates *NUAK1* and *Nuak2* expression at the transcriptional level. Translational inhibition resulted in accumulation of *NUAK1* and *Nuak2* mRNA in the absence of TGFβ (Fig. 2, *A* and *B*); NUAK1 or Nuak2 protein levels were markedly increased in the presence of TGFβ but disappeared after cycloheximide treatment, as expected (Fig. 2, *A* and *B*). SMAD2 phosphorylation, which verified active TGFβ signaling, was not influenced by cycloheximide, because TβRI phosphorylates a pre-existing pool of SMAD2 (Fig. 2, *A* and *B*).

Actinomycin D, which inhibits transcription, blocked TGFβ-induced *Nuak2* mRNA expression (Fig. 2*C*), supporting a transcriptional mechanism. Depletion of SMAD4 in AG1523 fibroblasts using transient siRNA transfection, blocked induction of NUAK1 protein by TGFβ (Fig. 2*D*), suggesting that TGFβ can enhance NUAK1 expression via a SMAD4-dependent pathway. Depleting *Smad4* mRNA in NMuMG cells blocked the induction of both *Nuak2* and *Gadd45*γ mRNAs by TGFβ (Fig. 2*E*); *Gadd45*γ is a known TGFβ-inducible gene (31) used as positive control. In SMAD4-null human breast carcinoma MDA-MB-468 cells, NUAK2 protein expression and weak inducibility by TGFβ were rescued upon reconstitution of SMAD4 in the cells (Fig. 2*F*). Inhibiting TβRI kinase activity with two independent chemical inhibitors (LY2157299 and SB505124) completely suppressed TGFβ-induced NUAK1 and NUAK2 levels in AG1523 cells (Fig. 2*G*). On the other hand, inhibiting MAPK ERK1/2 and p38 pathways with the inhibitors Cl-1040 (PD184352, MEK inhibitor) and SB203580, respectively, significantly but not completely blocked the inducibility of Nuak2 by TGFβ in NMuMG cells (Fig. 2*H*). Thus, TGFβ enhances *NUAK1* and *NUAK2* expression in a TβRI- and SMAD4-dependent manner, with additional contributions by the MAPKs in the case of *NUAK2*.

**Figure 3.**
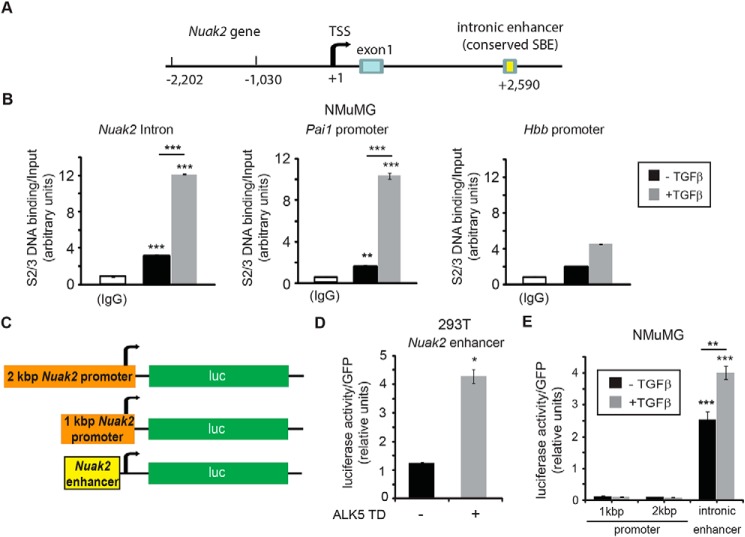
**Nuak2 is a direct TGFβ target gene.** *A*, cloning of the mouse *Nuak2* promoter and enhancer. Shown is a *schematic diagram* of the mouse *Nuak2* gene spanning the promoter sequences, the transcriptional start site (*TSS*), the first exon, and part of the first intronic sequence. *B*, ChIP assays using an antibody against endogenous SMAD2/SMAD3 (*S2/3*) and amplification of genomic sequences corresponding to the *Nuak2* intronic enhancer, the *Pai1* promoter, and the β-globin (*Hbb*) control region in NMuMG cells stimulated with or without 5 ng/ml TGFβ for 1 h. Control immunoprecipitations with mouse IgG are also shown as reference. The amount of PCR-amplified DNA signal after ChIP is normalized against the equivalent PCR signal of the input chromatin prior to immunoprecipitation, and the relative ratios are shown in the diagrams as average values determined from triplicate determinations with their corresponding S.E. (*error bars*). *C*, the genomic fragments, depicted in *A*, that were cloned into the luciferase reporter are shown relative to the luciferase (*luc*) cDNA in the corresponding constructs. *D*, luciferase assay from HEK 293T cells transiently transfected with the *Nuak2* enhancer construct and pcDNA3 control or constitutively active pcDNA3-ALK5TD. *E*, luciferase assay was performed using NMuMG cells transiently transfected with the *Nuak2* 1- or 2-kbp promoters and the *Nuak2* intronic enhancer constructs with or without TGFβ (5 ng/ml) stimulation for 17 h. Each independent experiment was repeated at least three times. *Asterisks* depict differences compared with respective controls or between the conditions indicated with *lines*: *, *p* < 0.05; **, *p* < 0.01; ***, *p* < 0.001.

**Figure 4.**
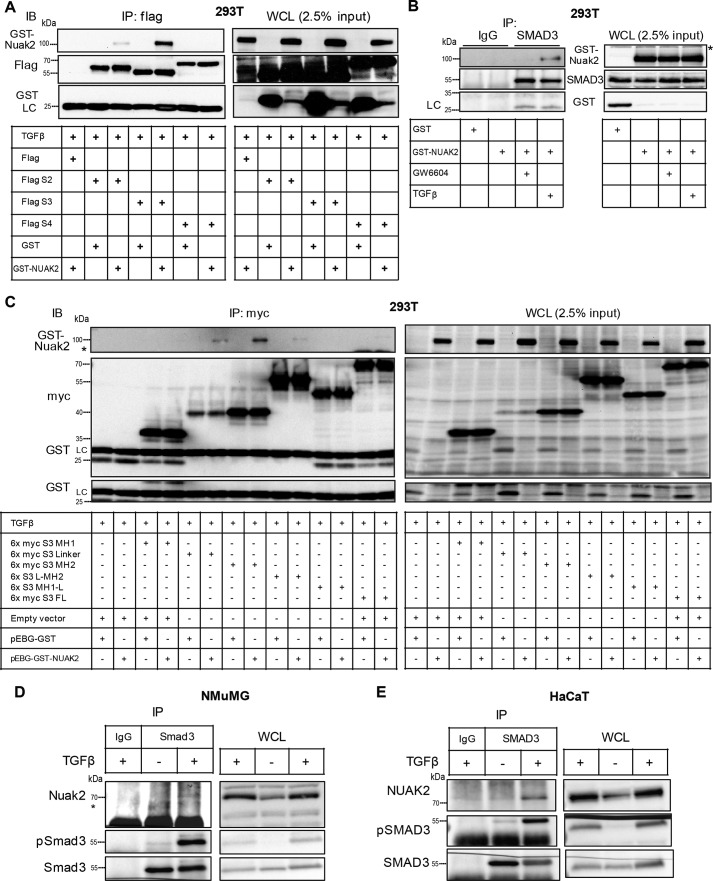
**NUAK2 physically interacts with SMAD3.** *A*, HEK 293T cells were co-transfected with FLAG-tagged SMAD2 (*S2*), SMAD3 (*S3*), or SMAD4 (*S4*) or the respective empty vector and GST-tagged NUAK2 or GST alone (0.5 μg of DNA/plasmid). After treatment with TGFβ (2.5 ng/ml) for 2.5 h, cell lysates were immunoprecipitated (*IP*) using FLAG-embedded agarose beads for 1 h, and proteins were eluted and subjected to SDS-PAGE. The corresponding whole-cell lysates (WCL; 2.5% of input used for immunoprecipitation) were analyzed in parallel. Proteins were detected by using NUAK2, FLAG, and GST antibodies. The immunoglobulin light chain (*LC*) is also marked. *IB*, immunoblotting. *B*, GST-tagged NUAK2 or GST alone was transiently transfected (1 μg of DNA/plasmid) in HEK 293T cells. Following either stimulation of cells with TGFβ (2 ng/ml) for 30 min or TβRI inhibitor GW6604 (3 μm) for 1 h to quench autocrine signaling, cell lysates were incubated overnight with SMAD3 antibody (0.5 μg) or its respective rabbit isotype (0.5 μg) as a negative control, followed by pulldown by protein A Dynabeads for 1 h. Immunoprecipitated proteins were eluted and resolved by SDS-PAGE, followed by immunoblotting for SMAD3, NUAK2, and GST. The anti-SMAD3 antibody never pulled down transfected GST alone but did immunoprecipitate GST-NUAK2 protein. Note that the protein band detected in the SMAD3 immunoprecipitation corresponds to the light chain of the anti-SMAD3 immunoglobulin (marked on the *left* of the immunoblot), which migrates slightly faster than the GST protein band shown in the *first lane* of the WCL. *C*, Myc-tagged domains of SMAD3 (MH1, MH2, linker (*L*), full-length (*FL*), MH1-L, and L-MH2) or Myc-tagged empty vector were co-transfected with GST-NUAK2 or GST alone (0.5 μg/DNA plasmid). After 1 day, cells were starved overnight with 2% FBS/DMEM, and the second day post-transfection, they were treated with TGFβ (2 ng/ml) for 2.5 h. Thereafter, cell lysates were immunoprecipitated with Myc-embedded agarose beads for 1 h. The samples were resolved by SDS-PAGE in parallel with their respective WCL, and proteins were identified by utilizing NUAK2, Myc, and GST antibodies. The immunoglobulin light chain is also marked. Note that the *middle* and *bottom* immunoblots are one and the same; the *bottom* is an exact duplication of part of the *middle* immunoblots that are exposed slightly longer to emphasize the lack of GST protein on the immunoprecipitation (*left part*) and its presence on the WCL (*right part*). *D*, NMuMG cells were treated with TGFβ (5 ng/ml) for 2 h, and then following immunoprecipitation with Smad3 or control antiserum, eluents together with WCL were resolved by SDS-PAGE, followed by Nuak2, Smad3, and pSmad3 immunoblotting. *E*, as in *D*, HaCaT cells were stimulated with TGFβ (5 ng/ml) for 2 h, and the cell lysates were incubated with SMAD3 antibody or control IgG overnight. Immunoprecipitated proteins were separated by SDS-PAGE, and NUAK2, SMAD3, and pSMAD3 were detected via immunoblotting. Molecular size markers in kDa are shown in every immunoblot.

Earlier genome-wide ChIP-Seq analysis (32) identified a SMAD2/3-binding region in the first *NUAK2* intron in HaCaT keratinocytes, and we could identify the homologous binding region in the mouse *Nuak2* gene as well (Fig. 3*A*). ChIP-qPCR analysis showed that TGFβ stimulation for 1 h potently induced SMAD2/3 binding to the *Nuak2* intronic enhancer region (Fig. 4*B*). SMAD2/3 binding to the plasminogen activator inhibitor 1 (*Pai1*) promoter and the hemoglobin B (*Hbb*) control promoter were included as positive and negative controls of the ChIP assay, respectively (Fig. 3*B*). We consider the weak TGFβ-induced binding of SMAD2/3 to the *Hbb* region as background nonspecific binding (Fig. 3*B*). Inspecting the promoter sequence up to 2 kbp upstream from the transcriptional start site (TSS), we could not identify any specific SMAD2/3 binding using ChIP (data not shown). We therefore cloned the mouse *Nuak2* intronic enhancer region into a luciferase construct containing a minimal promoter (Fig. 3*C*). Sustained TGFβ signaling due to transfection of a constitutively active TβRI (ALK5TD) resulted in an increase of luciferase activity compared with control (Fig. 3*D*), suggesting that the *Nuak2* intronic region has the potential to function as a TGFβ-inducible enhancer. Transfection of the same enhancer construct into NMuMG cells showed that TGFβ signaling enhanced its already high basal activity (Fig. 3*E*). Cloning of 1- or 2-kbp promoter regions of mouse *Nuak2* into luciferase constructs (Fig. 3*C*) did not yield any positive regulation by TGFβ (Fig. 3*E*), in agreement with the scan of the promoter region for the SMAD2/3-binding region via ChIP analysis. Thus, TGFβ signaling promotes *NUAK2* transcription minimally via a SMAD protein complex that associates with an enhancer in the first *NUAK2* intron. Whether a similar enhancer mediates inducibility of *NUAK1* to TGFβ remains to be examined.

### NUAK2 associates with SMAD3 and TβRI

A previous high-throughput screen for proteins that interact with TGFβ receptors or SMADs identified binding of NUAK2 to TβRI (33). Influenced by these results, we examined this possibility thoroughly. After co-expression of GST-NUAK2 together with FLAG-tagged SMAD2, SMAD3, or SMAD4, we observed strong complex formation between NUAK2 and SMAD3 and much weaker complex between NUAK2 and SMAD2 (Fig. 4*A*). GST-NUAK2 associated with endogenous SMAD3 when the cells were stimulated with TGFβ, whereas treatment with a potent TβRI kinase inhibitor (GW6604) resulted in weak, basal protein association (Fig. 4*B*). Probing for the GST tag of NUAK2 did not reveal any association between GST and SMAD3 (Fig. 4*B*).

SMAD3 is a modular protein composed of an N-terminal Mad homology 1 (MH1) domain that binds to DNA, contains a nuclear localization signal, and associates with transcription factors; a middle linker domain that is phosphorylated and ubiquitinated and regulates protein conformation and stability; and a C-terminal MH2 domain that becomes phosphorylated by TβRI in its very C-terminal diserine motif and interacts with transcription factors, performing a transcriptional activation function (2, 4, 6). Using deletion mutants of SMAD3, we could demonstrate that GST-NUAK2 formed complexes with the linker and MH2 domains of SMAD3, but not with the MH1 domain (Fig. 4*C*). Deletion mutants spanning the linker and MH2 domains and full-length SMAD3 protein resulted in much weaker protein complexes (Fig. 4*C*), suggesting that an exposed MH2 domain (possibly after C-terminal phosphorylation by TβRI) presents the highest affinity for NUAK2. At the endogenous level, association between Nuak2 and Smad3 could be demonstrated in mouse NMuMG (Fig. 4*D*) and between NUAK2 and SMAD3 in human HaCaT cells (Fig. 4*E*). In all cases, the association between endogenous proteins was exclusively TGFβ-dependent (Fig. 4, *D* and *E*).

**Figure 5.**
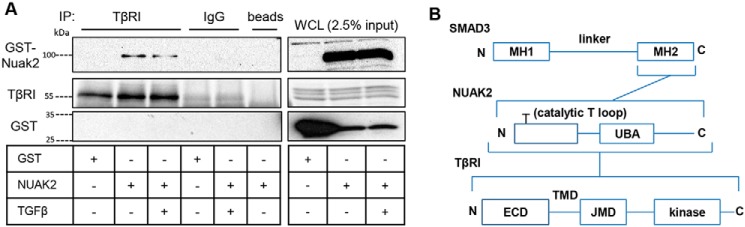
**NUAK2 associates with the TGFβ type I receptor.** *A*, GST-tagged NUAK2 or the respective empty vector was transiently transfected (1 μg of plasmid DNA in total) in HEK 293T cells. After incubation with TGFβ (2 ng/ml) for 30 min, cell lysates were incubated with TβRI antibody (2 μg), rabbit IgG (2 μg), or beads overnight, followed by incubation with protein A Dynabeads for 1 h. After washes, proteins were resolved by SDS-PAGE, and NUAK2, TβRI, or GST was detected by immunoblotting. WCL samples were analyzed concurrently. Molecular size markers in kDa are shown. *IP*, immunoprecipitation. *B*, diagram of physical interactions between NUAK2 and SMAD3 or TβRI. Shown is a *schematic representation* of SMAD3 domains containing the MH1, linker, and MH2 regions and a *schematic representation* of NUAK2 with Thr^208^ required for activation of protein kinase activity portrayed at the T loop in the N-terminal kinase domain, as well as the central ubiquitin-associated domain (*UBA*). The TβRI domains, extracellular (*ECD*), transmembrane (*TMD*), and juxtamembrane (*JMD*), containing the GS motif being phosphorylated by TβRII, and kinase, are depicted. *N* and *C*, the respective N and C termini of each protein. *Brackets* indicate domains that interact.

Using the same approach, we could also demonstrate that GST-NUAK2 forms complexes with TβRI, and this association did not change significantly after a brief stimulation of the cells with TGFβ (Fig. 5*A*). Control immunoglobulin or Sepharose beads generated clean background without nonspecific interactions (Fig. 5*A*). All of the above results confirm and significantly extend the original high-throughput findings (33) and provide evidence for complex formation between NUAK2 and two major components of TGFβ signaling, TβRI and SMAD3 (Fig. 5*B*).

### NUAK1 and NUAK2 differentially regulate matrix gene responses to TGFβ signaling

**Figure 6.**
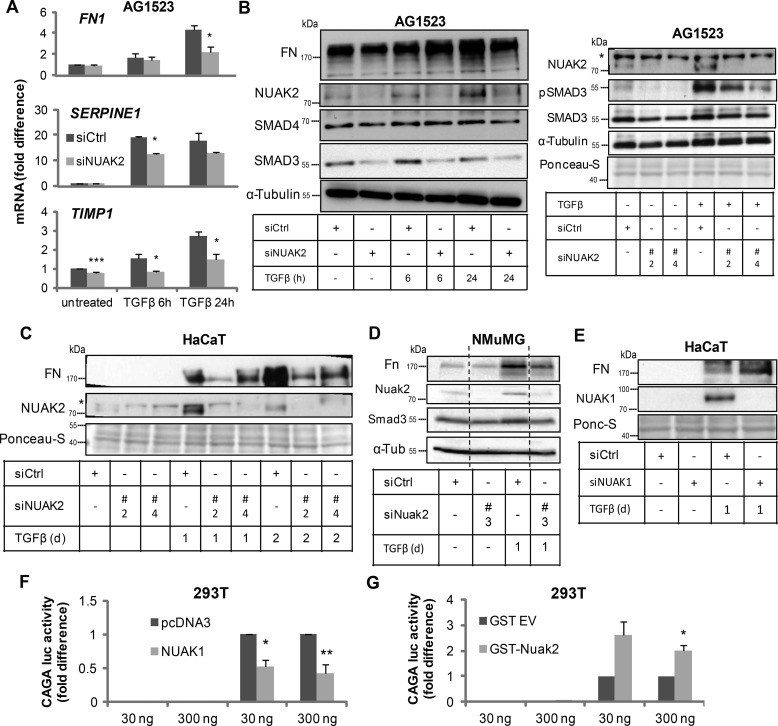
**NUAK1 and NUAK2 regulate TGFβ signaling.** *A*, relative mRNA levels of *SERPINE1*, *FN1* (fibronectin 1), and *TIMP1* were analyzed by real-time qRT-PCR. Values were normalized to *GAPDH* and are shown as -fold difference. AG1523 cells transfected with scrambled siRNA or siRNA targeting *NUAK2* were starved overnight in 0.01% FBS/DMEM and incubated with TGFβ (5 ng/ml) for different time periods. *B*, as in *A*, AG1523 cells post-siRNA transfection were starved and treated with TGFβ (5 ng/ml) for the indicated time periods. Samples were analyzed by immunoblotting for FN, NUAK2, SMAD3, SMAD4, and α-tubulin, used as a loading control. On the *right*, immunoblot analysis for NUAK2, pSMAD3, and SMAD3 was performed after transfecting AG1523 with two different siRNAs (#*2* and #*4*) targeting *NUAK2.* α-Tubulin and Ponceau-S staining serve as loading controls. A *star* shows nonspecific protein bands. Molecular size markers in kDa are shown. *C*, HaCaT cells transfected with control or two distinct *NUAK2* siRNAs (#*2* and #*4*) were either starved with 2% FBS/DMEM overnight and thereafter stimulated with TGFβ (5 ng/ml) for 24 h or treated directly with TGFβ (5 ng/ml) for 2 days. Samples were analyzed by immunoblotting for FN and NUAK2. Ponceau-S staining serves as a loading control. A *star* shows nonspecific protein bands. Molecular size markers in kDa are shown. *D*, NMUMG cells were subjected to transfection with scrambled siRNA or siRNA against *Nuak2* (*mm* #*3*); thereafter, they were stimulated with or without TGFβ (5 ng/ml) for 24 h, and immunoblot analysis against Nuak2, Fn, Smad3, and α-tubulin (α-Tub) was performed. Molecular size markers in kDa are shown. *Dotted lines* mark the removal of intermediate samples from the immunoblot. *E*, HaCaT cells transfected with scrambled siRNA or siRNA targeting *NUAK1*, were starved overnight in 2% FBS/DMEM. Three days after the first transfection, cells were treated with TGFβ (1 ng/ml) for 24 h. Respective immunoblotting of FN and NUAK1 proteins is depicted, and Ponceau-S (*Ponc-S*) staining served as loading control. Molecular size markers in kDa are shown. *F*, luciferase assay in HEK 293T cells that were transfected with CAGA_12_-luc and β-gal reporters together with the indicated expression vectors; 1 day post-transfection, cells were starved overnight in 2% FBS/DMEM and were subjected to TGFβ stimulation (1 ng/ml) for 24 h. Luciferase activity was measured and normalized to β-gal. Values are depicted as -fold difference. All *graph bars* are shown as average ± S.E. (*error bars*) based on at least three independent experiments. *Asterisks* illustrate significant differences between the conditions indicated and respective control: *, *p* < 0.05; **, *p* < 0.01; ***, *p* < 0.001.

To assess the function of NUAK1 and NUAK2 induction in response to TGFβ signaling, and influenced by the ability of NUAK2 to associate with SMAD3 and TβRI, we knocked down NUAK2 using siRNA in the AG1523 fibroblasts and examined first the potency of regulation of established TGFβ-induced genes, such as the extracellular matrix genes *SERPINE 1* (plasminogen activator inhibitor 1, *PAI1*), fibronectin 1 (*FN1*), and TIMP metallopeptidase inhibitor 1 (*TIMP1*; Fig. 6*A*). TGFβ induced *FN1*, *SERPINE1*, and *TIMP1* levels in a time-dependent manner; however, knockdown of NUAK2 reduced the potency of induction of these genes by TGFβ (Fig. 6*A*). FN protein expression analysis gave similar results, especially at late time points, when the levels of endogenous NUAK2 induced by TGFβ in control cells were high (Fig. 6*B*). The same result was corroborated in HaCaT keratinocytes, where two individual and distinct siRNAs targeting human NUAK2 effectively reduced the inducibility of FN by TGFβ (Fig. 6*C*). The two distinct and single siRNA oligonucleotides verified that the results obtained based on siRNA pools used in the previous experiments (Fig. 6, *A* and *B*) were not prone to off-target effects. To bypass even more the possibility of off-target effects, we analyzed FN protein expression in mouse NMuMG cells after transfection of individual siRNAs targeting mouse Nuak2 and confirmed again the positive role of endogenous Nuak2 on FN inducibility by TGFβ (Fig. 6*D*).

Unexpectedly, control experiments, where the steady-state levels of SMAD proteins were monitored, revealed that silencing of NUAK2 in AG1523 fibroblasts had a strong impact on the level of SMAD3, but not of SMAD4 (Fig. 6*B*), suggesting that the interaction between NUAK2 and SMAD3 (Fig. 4) may play a positive role in preserving SMAD3 protein stability. Repeating this experiment with the two individual siRNAs in the fibroblasts corroborated the observations and confirmed down-regulation of phosphorylated and of total SMAD3 levels upon NUAK2 depletion (Fig. 6*B*). The same result was further consolidated in mouse NMuMG cells using an additional and distinct siRNA (Fig. 6*D*). These results suggested that TGFβ-induced NUAK2 may function as a positive regulator of sustained (long-term) TGFβ signaling responses. Under the above conditions, NUAK1 silencing in HaCaT cells enhanced the response of endogenous FN to TGFβ stimulation (Fig. 6*E*), exhibiting an opposite phenotype compared with the NUAK2 knockdown (Fig. 6*C*) in the same cells.

Finally, to provide more direct evidence for the negative (NUAK1) or positive (NUAK2) role that these two kinases play in TGFβ signaling, we employed a luciferase reporter construct that is sensitive to the transcriptional activity of the SMAD3/SMAD4 complex, CAGA_12_-luc (Fig. 6, *F* and *G*). Transient expression of exogenous NUAK1 in HEK 293T cells suppressed the CAGA_12_-luc transcriptional response to TGFβ (Fig. 6*F*), whereas transient expression of NUAK2 in the same cells strongly enhanced the promoter response (Fig. 6*G*), suggesting that the two protein kinases have an impact on the transcriptional activity of the SMAD3/4 complex. These data therefore support a role of NUAK1 as a negative mediator, and of NUAK2 as a positive mediator, of TGFβ signals.

### Regulation of cytostatic and epithelial–mesenchymal transition (EMT) responses by NUAK kinases

**Figure 7.**
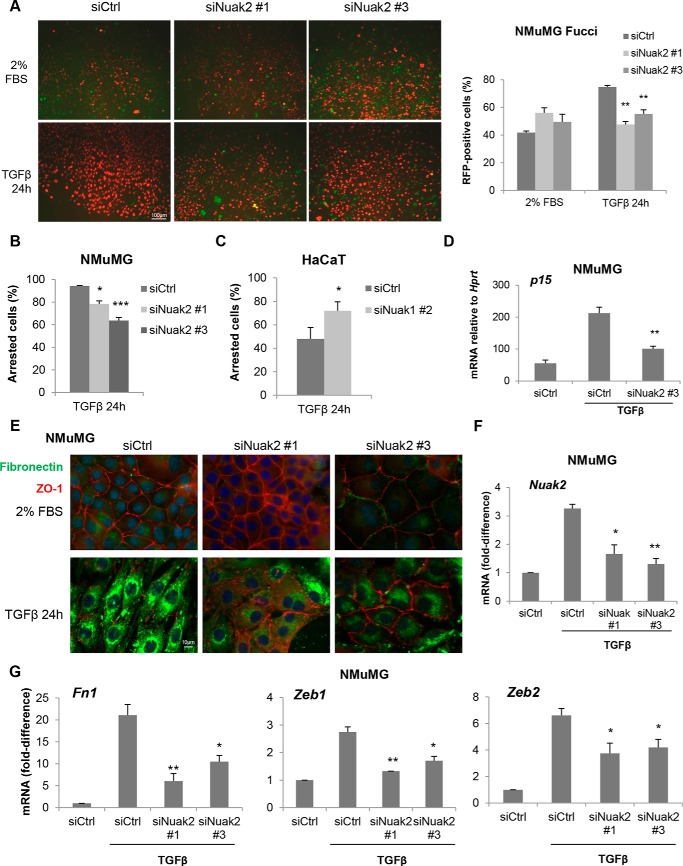
**Regulation of epithelial cytostasis and EMT by the NUAKs.** *A*, live NMuMG-Fucci cell imaging indicating *green* (S/M/G_2_ phase) and *red* (G_1_/G_0_ phase) nuclei in cells transiently transfected with individual and distinct siRNAs targeting mouse *Nuak2* and stimulated with 5 ng/ml TGFβ for 24 h. *Bars*, 100 μm. Quantification of the RFP-positive nuclei in each condition is described under “Experimental procedures.” *B* and *C*, NMuMG (*B*) or HaCaT (*C*) cells transfected with nontargeting control or individual siRNAs targeting mouse *Nuak2* (*B*) or human NUAK1 (*C*) and treated with TGFβ (1 ng/ml) for 24 h. Values were normalized to 2% FBS/DMEM-treated samples for each siRNA sample. *D*, *F*, and *G*, relative mRNA levels of *p15* (*D*), *Nuak2* (*F*), *Fn1*, *Zeb1*, and *Zeb2* (*G*) were analyzed by real-time qRT-PCR. Values were normalized to *Gapdh* and are shown as -fold difference. NMuMG cells transfected with nontargeting control or individual and distinct siRNAs targeting mouse *Nuak2* were incubated with TGFβ (5 ng/ml) for 24 h. *E*, fibronectin (*green*) and ZO-1 (*red*) immunofluorescence microscopy along with nuclear 4′,6′-diamidino-2-phenylindole (*blue*) staining of NMuMG cells after individual and distinct *Nuak2* or negative control siRNA transfection. Cells were incubated with TGFβ (5 ng/ml) for 24 h. Representative photomicrographs are shown. *Bars*, 10 μm. All *graph bars* are shown as average ± S.E. (*error bars*) based on at least three independent experiments. *Asterisks* illustrate significant differences between the conditions indicated and respective control: *, *p* < 0.05; **, *p* < 0.01; ***, *p* < 0.001.

Another well-established TGFβ-mediated physiological response is the cell cycle arrest of epithelial cells, and NMuMG epithelial cells and HaCaT keratinocytes have been valuable cell models in such studies (6). We first used the NMuMG-Fucci cell model that tracks the phases of the cell cycle based on accumulation of fluorescent probes (GFP mAG fused to geminin generates green color in the nuclei of cells during S/M/G_2_ phases, and red fluorescent protein mKO2 fused to Cdt1 generates red color in the nuclei of cells during the G_1_/G_0_ phases (7, 9)). TGFβ stimulation for 24 h arrested most of the cells (75–80%) in G_1_ (red nuclei; Fig. 7*A*). Silencing Nuak2 with two independent siRNAs significantly suppressed the TGFβ-dependent cell cycle arrest (Fig. 7*A*). As a verification of the above results, thymidine incorporation assays in parental NMuMG cells, where TGFβ suppresses incorporation up to 90%, revealed that silencing Nuak2 with the same individual mouse siRNAs significantly suppressed the TGFβ-induced growth arrest (Fig. 7*B*). We then verified the impact of NUAK1 by testing human HaCaT cells. TGFβ stimulation significantly reduced the number of HaCaT cells, which actively incorporated thymidine (S-phase cells), and correspondingly, the number of cell cycle arrested cells was measurable (30–35%, Fig. S2). Silencing NUAK1 with an siRNA pool doubled the number of cell cycle–arrested cells (Fig. S2), and silencing with an independent individual siRNA had a comparable effect (Fig. 7*C*), confirming that NUAK1 plays a negative role by limiting one of the most characteristic physiological responses to TGFβ. Because key mediators of the cytostatic response to TGFβ are cyclin-dependent kinase inhibitors, such as *p15* (*CDKN2B*), we also assessed p15 mRNA expression in NMuMG cells; as expected, TGFβ-induced *p15* mRNA levels were strongly suppressed upon Nuak2 silencing (Fig. 7*D*).

We then continued the analysis of EMT, a biologically important response of epithelial cells to TGFβ (1, 6). NMuMG cells are an excellent model for this response (6), and silencing of endogenous Nuak2 by two independent siRNAs blocked the EMT, assessed microscopically as the loss of tight junctions (ZO-1 protein loss) and the strong gain of intra- and extracellular fibronectin deposition (Fig. 7*E*), two hallmark molecular attributes of the EMT (6). Silencing efficiency of Nuak2 in NMuMG cells was assessed during the EMT assays (Fig. 7*F*) along with mRNA analysis of additional EMT markers, including *Fn1* (fibronectin 1) mRNA along with two major pro-EMT transcription factors, *Zeb1* and *Zeb2* (Fig. 7*G*). Once again, Nuak2 contributed positively to the TGFβ-mediated induction of mRNA levels for all three genes (Fig. 7*G*). Thus, the regulation of NUAK kinases seems to regulate multiple physiological responses to TGFβ.

### Mesenchymal cell responses to TGFβ are differentially regulated by NUAK1 and NUAK2

**Figure 8.**
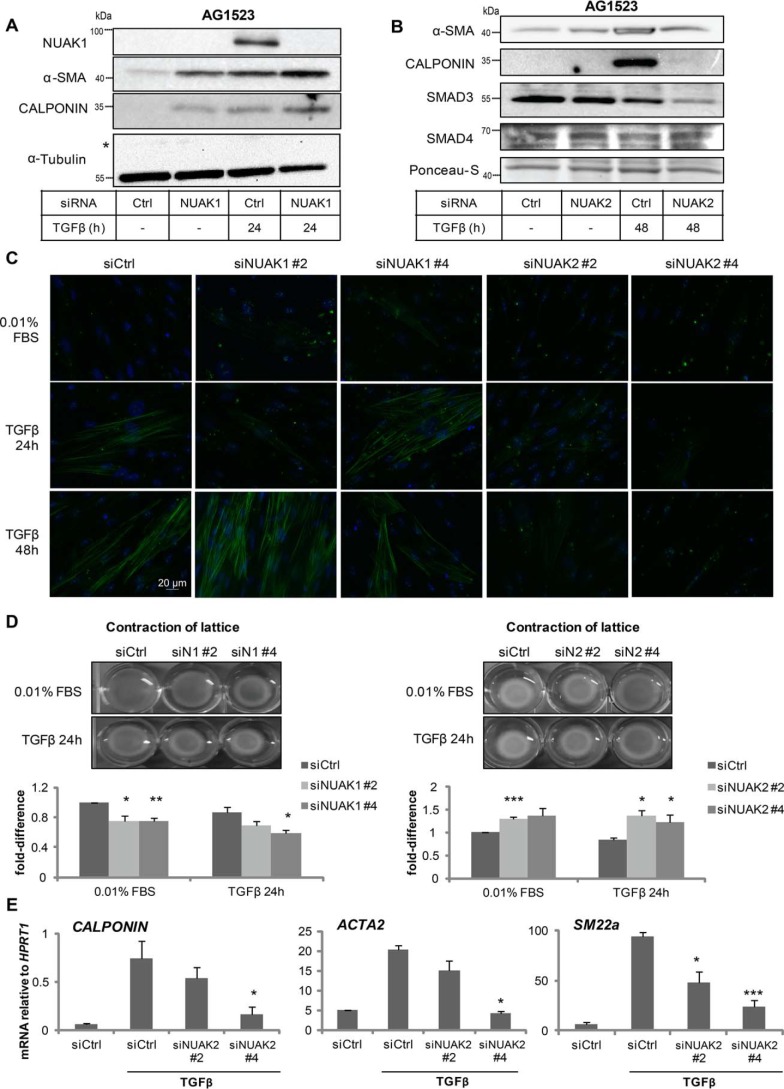
**Regulation of myofibroblast differentiation by the NUAKs.** *A* and *B*, AG1523 cells were subjected to double transfection with negative control or siRNA targeting *NUAK1* (*A*) or *NUAK2* (*B*) and treated with TGFβ (1 ng/ml) for 24 h. Samples were subjected to immunoblotting for NUAK1, αSMA, calponin, and α-tubulin, which serves as a loading control (*A*), or for NUAK2, αSMA, calponin, SMAD3, and SMAD4, with Ponceau-S staining included as a loading control (*B*). A *star* shows nonspecific protein bands. Molecular size markers in kDa are shown. *C*, αSMA immunofluorescence microscopy of AG1523 cells after *NUAK1*, *NUAK2*, or negative control siRNA transfection using individual and distinct siRNAs. Cells were starved in 0.01% FBS/DMEM and incubated with TGFβ (1 ng/ml) as indicated. Representative photomicrographs are shown. *Bar*, 20 μm. *D*, collagen gel contraction assay with AG1523 cells transfected with control or individual and distinct *NUAK1* siRNAs (*left panels*) or individual and distinct *NUAK2* siRNAs (*right panels*). Two days after the first transfection, TGFβ (5 ng/ml in 0.01% FBS/DMEM) was added, and the contracted lattice surface was measured 24 h post-treatment. Surface area was measured by using the ImageJ software and is illustrated in the corresponding graphs. Representative pictures of contracted lattices are shown. Bar graphs show average values derived from triplicate determinations and the corresponding S.E. (*error bars*). Each independent experiment was repeated at least three times. *E*, mRNA levels of calponin, *ACTA2*, and *SM22*α from the samples used in *C* and *D*. After performing real-time qRT-PCR, values were normalized to *HPRT1* mRNA. *Asterisks* illustrate significant differences between the conditions indicated and respective control: *, *p* < 0.05; **, *p* < 0.01; ***, *p* < 0.001.

The previous biological assays were all based on epithelial cells. We then shifted our attention to mesenchymal cell responses (34). Fibroblasts respond potently to TGFβ and synthesize a new contractile cytoskeletal machinery characterized by α-smooth muscle actin (αSMA) and associated proteins, including calponin and SM22α (34). Silencing endogenous NUAK1 in the AG1523 fibroblasts induced αSMA and calponin, roughly to the same extent as TGFβ stimulation for 24 h (Fig. 8*A*). Silencing of NUAK1 combined with TGFβ stimulation enhanced the αSMA and calponin protein responses even further (Fig. 8*A*). In the same cell model, NUAK2 exhibited the inverse behavior, as expected from all previous results. Silencing endogenous NUAK2 in the fibroblasts reduced the inducibility of αSMA and calponin by TGFβ and also reduced the steady-state levels of SMAD3 (Fig. 8*B*).

These results were also evident after immunofluorescence microscopy of the fibroblasts, whereby intense αSMA-positive microfilaments could be observed upon TGFβ stimulation in the presence of NUAK1 silencing using two independent siRNAs (Fig. 8*C*). The strong αSMA microfilament network induced by TGFβ became unstable and fragmented after NUAK2 silencing using two additional and distinct siRNAs (Fig. 8*C*). Contraction of extracellular collagen type I gels by differentiating myofibroblasts could be measured in response to TGFβ; NUAK1 silencing using the individually validated siRNAs enhanced basal and TGFβ-induced gel contractility (Fig. 8*D*). On the other hand, when NUAK2 levels were significantly reduced by independent siRNAs, there was reduced basal and TGFβ-induced gel contractility (Fig. 8*D*). Gene expression analysis after endogenous NUAK2 silencing with the independent siRNAs revealed that calponin, *ACTA2*/α*SMA*, and *SM22*α mRNA levels were decreased upon NUAK2 silencing (Fig. 8*E*). Thus, the NUAKs provide signals that balance the ability of fibroblasts to differentiate to contractile myofibroblasts in response to TGFβ.

Another mesenchymal cell model that we examined was mouse C2C12 myoblasts that can differentiate to myotubes upon starvation *in vitro*, a differentiation process potently blocked by TGFβ signaling (35). Previous studies have established myosin heavy chain (myosin HC) and the transcription factor myogenin as key target genes of TGFβ/Smad3 signaling in these myoblasts (35). Both myosin HC and myogenin levels were induced upon C2C12 differentiation, and TGFβ signaling suppressed this response (Fig. S3). Silencing endogenous Nuak1 by individual siRNAs enhanced the suppressive response, whereas Nuak2 silencing exhibited a partial but significant resistance to myosin HC and myogenin down-regulation by TGFβ (Fig. S3). In summary, the above results establish the two sister kinases as potent negative (NUAK1) and positive (NUAK2) contributors of TGFβ signaling in at least two mesenchymal differentiation models.

## Discussion

In this paper, we demonstrate that TGFβ signaling induces *NUAK1* and *NUAK2* transcription via SMAD and MAPK activity (Figs. 1–3). NUAK2 protein interacts with SMAD3 and TβRI (Figs. 4 and 5), suggesting a possible role in controlling the output of TGFβ signaling. Indeed, mainly silencing, but also experiments where NUAK1 and NUAK2 were overexpressed in various cell types, established that NUAK1 is a negative mediator of TGFβ signaling, whereas NUAK2 positively contributes to the signal transduction by this cytokine (Figs. 5–7). The functions of the two NUAK kinases seem to affect basic TGFβ signaling, as multiple epithelial and mesenchymal cell responses are impacted by genetic perturbation of these kinases (Figs. 6–8). The impact of each NUAK kinase in differentiating myofibroblasts establishes NUAK1 as an anti-fibrotic factor and NUAK2 as a pro-fibrotic factor (Fig. 8). These data propose that the transcriptional induction of the two NUAK proteins generates two signaling branches, one negative and another positive.

NUAK1 and NUAK2, together with SIK (7, 9), are members of the AMPK family of kinases, whose expression is directly regulated by TGFβ signaling. Each of these kinases participates in different molecular processes; SIK negatively regulates TGFβ receptor signaling, whereas NUAK1 and NUAK2 regulate SMAD transcriptional activity and SMAD3 protein levels. Interestingly, these kinases also require activation by their upstream regulator LKB1, and furthermore, LKB1 provides feedback regulation to TGFβ signaling, by phosphorylating SMAD4 (36). This highlights an intimate cross-talk between TGFβ, LKB1, and specific AMPKs, which is exemplified by studies in LKB1 knockout mice; LKB1 loss leads to ineffective synthesis and secretion of TGFβ ligands, thus leading to the development of hamartomatous polyps, a hallmark of the human genetic syndrome Peutz–Jeghers, which predisposes to intestinal cancer development (37).

The transcriptional induction of *NUAK1* and *NUAK2* by TGFβ is direct and involves SMAD and MAPK activities; in the case of *NUAK2*, an enhancer sequence resides in the first intron of the gene, which binds the SMAD complex and, upon molecular cloning, confers inducibility to TGFβ (Fig. 3). Whether a similar enhancer sequence resides in the vicinity of the *NUAK1* gene remains to be explored. Because SMAD complexes associate with chromatin via interaction with DNA and sequence-specific transcription factors, transcriptional regulation of *NUAK1* and *NUAK2* probably depends on additional SMAD-interacting cofactors that remain to be elucidated and that possibly receive inputs by TGFβ-induced MAPKs.

The transcriptional induction of NUAK2 by TGFβ is further linked to the ability of the protein kinase to form physical associations with SMAD3 and the TβRI (Figs. 4 and 5). These results nicely confirm earlier high-throughput findings using the LUMIER proteomic platform for the identification of proteins interacting with TGFβ receptors and SMAD proteins (33). Our findings establish firmly this interaction using complementary biochemical assays and further map the interaction between the MH2 domain of SMAD3 and NUAK2 (Fig. 4*C*). The fact that NUAK2 interacts with TβRI and one of its immediate substrates, SMAD3, generates the hypothesis that NUAK2 may promote the interaction between TβRI and SMAD3, facilitating its C-terminal phosphorylation by TβRI. This raises the possibility that NUAK2 might phosphorylate either TβRI or SMAD3. Attempts to test this hypothesis by *in vitro* phosphorylation assays did not give positive results (data not shown).

Silencing endogenous NUAK2 significantly down-regulated SMAD3 protein levels in various cell types (Fig. 6). It is possible that NUAK2 stabilizes SMAD3, thus mediating a positive role during TGFβ signaling (see below). Furthermore, interaction assays similar to those performed for NUAK2 (Figs. 4 and 5) failed to demonstrate an association between NUAK1 and SMAD3 or TβRI (data not shown). Despite the above caveat, all functional experiments so far converge on a model whereby NUAK1 and NUAK2 significantly regulate the output of TGFβ signaling (Figs. 6–8). The impact of NUAK2 on TGFβ signaling agrees with findings where NUAK2 enhances TGFβ signaling in hepatocytes infected with hepatitis C virus, thus promoting liver fibrosis (18). The regulation of CAGA_12_-luc reporter transcriptional activity by NUAK1 and NUAK2 (Fig. 6, *F* and *G*) strongly suggests that these two protein kinases regulate the function of the SMAD3/SMAD4 complex, either in a direct manner (*e.g.* based on the association of NUAK2 with SMAD3) or indirectly via regulation of enzymes that control the stability and activity of nuclear SMADs (2).

As expected from the above impact of NUAK1 and NUAK2 on TGFβ signaling, these two protein kinases regulate TGFβ-induced cytostasis, EMT, myofibroblast differentiation, and contractility and suppression of myoblast differentiation (Figs. 7 and 8). NUAK1 and NUAK2 appear as proteins that “sense” the differentiation state of fibroblasts and mediate important functions during the myofibroblast switch. Such functions may involve the established phosphorylation and regulation of proteins of the myosin light chain system (17, 23). However, the multiple responses to TGFβ in epithelial and mesenchymal cells, some positive (EMT and myofibroblast differentiation) and some negative (*e.g.* suppression of epithelial cell cycle and of myocyte differentiation), suggest that the NUAKs may direct their activity toward the TGFβ pathway, independent of biological context. A prediction worth testing is that elimination of both NUAK1 and NUAK2 simultaneously might result in neutral effects on TGFβ signaling.

*In vivo* functions of Nuak1 and Nuak2 have been analyzed in mice. *Nuak1* knockout leads to embryonic lethality due to defects in ventral body wall closure, whereas *Nuak2* knockout causes partial exencephaly (38). Double-knockout mutant mice generate facial clefting, spina bifida, and a stronger exencephaly (relative to single *Nuak2* knockout) phenotypes (38). Facial clefting resembles a phenotype revealed in the *TGF*β*3* knockout mouse, which may be compatible with a positive role of Nuak2 downstream of TGFβ signaling, as demonstrated in this paper. Nuak1 can phosphorylate and stabilize Tau in neurons and, thus, contributes to regenerative deterioration of the brain in mice (39). The heterozygous *Nuak1* knockout/WT mouse exhibits haploinsufficiency that partially rescues neuronal deterioration caused by Tau accumulation (39). Because TGFβ signaling protects from brain degeneration, it is possible that the negative impact that NUAK1 has on TGFβ signaling may reflect a mechanism of progressive deterioration of TGFβ actions during neurodegeneration. Furthermore, the NUAKs are implicated in muscle homeostasis *in vivo* but do not show overt defects of muscle differentiation when knocked out in mice (16, 40). Our evidence suggests involvement of the two NUAKs in C2C12 myoblast differentiation and its inhibition by TGFβ (Fig. S3). Whether suppression of insulin signaling and glucose uptake in *Nuak1*-mutant muscle (40) or positive regulation of myocyte survival and muscle mass maintenance during aging by Nuak2 (16) reflect processes controlled by TGFβ signaling, among other key pathways, remains to be examined.

We propose that the balance between the seemingly opposite roles of NUAK1 and NUAK2 on TGFβ signaling can play important roles in epithelial and mesenchymal cell physiology. The spectrum of actions of NUAK1 and NUAK2 may range from the control of gene expression to the regulation of the cell cycle and cell differentiation, important cellular properties that define adult tissue homeostasis and diseases, such as tissue fibrosis and cancer.

## Experimental procedures

### Cell culture, transfections, and adenoviral infections

All original cell lines were obtained from ATCC (Manassas, VA). Mouse mammary epithelial NMuMG cells and their clone 18 (41) and NMuMG-Fucci (7, 9), mouse C2C12 pluripotent cells, immortalized human keratinocytes HaCaT, primary human skin fibroblasts AG1523 used up to passage 20, cervical carcinoma HeLa cells, human lung adenocarcinoma A549 cells, human breast carcinoma MDA-MB-468 cells, and human embryonic kidney 293T cells were grown in DMEM containing 10% (or 15% in the case of C2C12) fetal bovine serum (FBS) (Biowest, Biotech-IgG AB, Lund, Sweden), penicillin and streptomycin, and 5 mm l-glutamine. Immortalized normal human mammary epithelial cells MCF10A and their Ras-transformed premalignant MCF10AneoT (MII) derivatives were cultured in DMEM/F-12 (Gibco, Life Technologies Europe BV, Stockholm, Sweden) supplemented with 5% horse serum (Biowest, Biotech-IgG AB, Lund, Sweden), 100 ng/ml cholera toxin (Sigma-Aldrich Sweden AB, Stockholm, Sweden), 20 ng/ml epidermal growth factor (Upstate, Millipore AB, Solna, Sweden), 0.5 μg/ml hydrocortisone (Sigma-Aldrich Sweden AB), and 10 μg/ml insulin (Sigma-Aldrich Sweden AB). Cells were maintained at 5% CO_2_ in a humidified atmosphere at 37 °C. TGFβ1 (PeproTech EC Ltd. Nordic (Stockholm, Sweden) and BIOSOURCE Inc. (Dacula, GA)), abbreviated as TGFβ, was used at concentrations as indicated in each experiment, spanning from 1 to 5 ng/ml.

Scrambled (D-001810-10-50), hsNUAK1 (LU-004931-01), hsNUAK2 (L-005374-00), hsSMAD4 (L-003902-00) SMARTpool siRNAs, individual hsNUAK1 nr 2 (J-004931-10), hsNUAK1 nr 4 (J-004931-12), hsNUAK2 nr 2 (J-005374-09), and hsNUAK2 nr 4 (J-005374-11) were used. All siRNAs were purchased from Dharmacon (Thermo Fisher Scientific, Gothenburg, Sweden) and were transiently transfected twice on two consecutive days at 20 nm each, using Silentfect (Bio-Rad Laboratories AB, Sundbyberg, Sweden), into cells cultured in 5% FBS/DMEM in the absence of antibiotics, 72 h prior to TGFβ stimulation. Single transfection of 100 nm mmNuak2 (L-051199-00), mmSmad4 (L-040687-00), or nontargeting (D-001810-10) ON-TARGETplus SMART pool siRNAs from Dharmacon (Thermo Fisher Scientific) was performed in NMuMG clone 18 cells. Individual mmNuak1 nr 3 (J-063024-07), mmNuak2 nr 1 (J-051199-05), and mmNuak2 nr 3 (J-051199-07) siRNAs, also from Dharmacon (Thermo Fisher Scientific), were transiently transfected into subconfluent C2C12 cell cultures on two consecutive days, at a final concentration of 50 nm each time, in DMEM plus 5% FBS. Two days after the first transfection, differentiation medium (DMEM plus 2% horse serum) was added in the presence or absence of TGFβ (5 ng/ml) for 3 days. Then fresh medium was added for additional 3 days (6 days in total). Luciferase constructs were transfected into HEK 293T cells using Fugene HD (Roche AB, Solna, Sweden) according to the manufacturer's instructions.

For co-immunoprecipitation experiments, mammalian pcDNA3 empty vector or pcDNA3-FLAG-tagged SMAD2, SMAD3, or SMAD4; pcDNA3–6myc-tagged domains of SMAD3 (MH1, MH2, linker (L), full-length (FL), MH1+L, and L+MH2); or pcDNA3–6myc-tagged empty vector have already been described (42, 43). Human *NUAK2* cDNA was cloned in the pEBG2t vector as a 2-kbp insert in Spe1-Spe1 restriction sites giving rise to GST-tagged NUAK2 and was provided by James C. Hastie (London, UK). All of the constructs with their corresponding controls were co-transfected to HEK 293T cells as 0.5 μg of plasmid DNA, unless stated otherwise, by using Fugene HD (Roche AB), following the manufacturer's instructions.

Adenoviruses expressing β-gal (Adex-lacZ) and human SMAD4 (Adex-SMAD4) have been described previously (44). Briefly, subconfluent MDA-MB-468 cells were trypsinized, counted, and reseeded in 0.01% FBS-containing medium in 24-well plates at a concentration of 60,000 cells/well and used in triplicates per condition. While still in suspension, adenoviral constructs were added to the medium. Twenty-four hours postinfection, cells were treated with TGFβ for the time periods indicated in the figures, and protein expression or luciferase activity was measured as described below.

### Antibodies and chemicals

Anti-NUAK2 antibody was purchased from Sigma-Aldrich Sweden AB; anti-phosho(Ser-465/Ser-467)-SMAD2 antibody was homemade; anti-SMAD2 antibody was from Epitomics, Cell Marque/Sigma-Aldrich Sweden; anti-α-tubulin, anti-β-actin, anti-GST, anti-Myc, and anti-TβRI V22 were from Santa Cruz Biotechnology Inc. (Dallas, TX); anti-SMAD2/3 antibody and anti-PAI-1 (plasminogen activator inhibitor 1) were from BD Bioscience AB, Stockholm, Sweden; anti-NUAK1, anti-phospho-SMAD3, anti-SMAD3, anti-SMAD4, anti-phospho-p44/42 MAPK (ERK1/2-Thr^202^/Tyr^204^), and anti-Snail (catalog no. 3789) antibodies were from Cell Signaling Technology (Leiden, The Netherlands); anti-myosin heavy chain antibody clone A4.1025 and anti-myogenin were from Merck/Millipore (Darmstadt, Germany).

Cycloheximide (Sigma-Aldrich Sweden AB) was used at a concentration of 40 μg/ml, and actinomycin D (Sigma-Aldrich Sweden AB) was used at 4 μg/ml. TβRI kinase inhibitor GW6604 was used at a final concentration of 3 μm and was synthesized by the Ludwig Cancer Research Ltd. TβRI kinase inhibitor LY2157299 (Cayman Chemical Co., Stockholm, Sweden) was used at a final concentration of 5 μm, whereas TβRI kinase inhibitor SB505124 (Sigma-Aldrich Sweden AB) was applied at a concentration of 2.5 μm. MEK kinase inhibitor Cl-1040 (PD184352), used at 0.5 μm, and p38 MAPK inhibitor SB203580, used at 10 μm, were from Calbiochem, Merck (Darmstadt, Germany). Chemical inhibitors or DMSO (vehicle) were added to cells starved overnight, 1 h prior to TGFβ stimulation.

### Immunoblotting and co-immunoprecipitation

Cells were lysed in radioimmune precipitation assay buffer containing 50 mm Tris-HCl, pH 8, 150 mm NaCl, 1% NP-40, 0.1% SDS, 0.5% sodium deoxycholate, supplemented with complete protease inhibitor mixture (Roche Diagnostics, Bromma, Sweden) and phosphatase inhibitors (1 mm sodium orthovanadate, 50 mm sodium fluoride), incubated for 30 min on ice including occasional vortexing, centrifuged at 13,000 rpm, and boiled in sample buffer containing 5% β-mercaptoethanol as a reducing agent and 2% SDS as a denaturing agent. Samples were subjected to SDS-PAGE, followed by wet transfer to nitrocellulose membranes (Amersham Biosciences Protran 0.45 NC, GE Healthcare, Uppsala, Sweden) and blocking (5% BSA in Tris-buffered saline, 0.1% Tween 20) for 1 h at room temperature. Primary antibodies were incubated overnight at 4 °C. Secondary antibodies were incubated for 1 h at room temperature before detection with chemiluminescence substrate (Santa Cruz Biotechnology) and X-ray films (Fujifilm Nordic AB, Stockholm, Sweden).

For co-immunoprecipitation assays, cells were lysed in a buffer consisting of 0.5% Triton X-100, 11.5 mm sodium deoxycholate, 20 mm Tris-HCl, pH 7.4, 150 mm NaCl, 10 mm EDTA, and complete protease inhibitor mixture from Roche Diagnostics for 30 min on ice; the cell pellet was removed after centrifugation at 13,000 rpm for 15 min at 4 °C. Cell lysates were precleared for 1 h with 10 μl of protein A Dynabeads end-over-end. Subsequently, they were incubated with mouse anti-FLAG-M2 F-3165 (Sigma-Aldrich Sweden AB) or anti-Myc (Thermo Fisher Scientific) embedded agarose beads (30-μl final volume PBS/slurry, 1:1) for 1 h at 4 °C. For semi-endogenous immunoprecipitations, 3 μg of anti-TβRI V22 or normal anti-rabbit IgG (ab46540, Abcam, Cambridge, UK) were used, and for SMAD3 co-immunoprecipitations, 3 μg of specific antibody (ab28379, Abcam) or an equivalent amount of anti-rabbit IgG as a negative control were used. On the next day, 30 μl of protein A Dynabeads were added to the lysates for 1 h at 4 °C. After five washes with lysis buffer, the immunocomplexes were resolved by SDS-PAGE and immunoblotted with antibodies, as described in the figure legends.

### Chromatin immunoprecipitation (ChIP)

NMuMG cells were grown to 80–90% confluence in 15-cm plates and stimulated with 5 ng/ml TGFβ for 1 h, prior to cross-linking with 1% formaldehyde via incubation on a shaking platform for 10 min at room temperature. Cross-linked cells were washed with ice-cold PBS, and cell pellets were resuspended in 1.5 ml of lysis buffer (50 mm Tris-HCl, pH 8.0, 10 mm EDTA, 1% SDS, supplemented with protease inhibitor mixture (Roche Diagnostics)). Total cell lysate was sonicated in a water-bath Diagenode Bioraptor sonicator with 30-s pulses for 15 min at high frequency to obtain short DNA fragments. The lysate was subsequently centrifuged at 14,000 rpm in 4 °C for 10 min. ChIP was performed overnight at 4 °C with 10 μg of mouse monoclonal anti-SMAD2/3 (BD Bioscience AB) or 10 μg of nonspecific preimmune mouse immunoglobulin (homemade), together with magnetic beads (Dynabeads M280, Invitrogen, Thermo Fisher Scientific) and dilution buffer (20 mm Tris-HCl, pH 8.0, 2 mm EDTA, 1% Triton X-100, 150 mm NaCl, and protease inhibitor mixture (Roche Diagnostics) in a total volume of 15 ml (sonicated cell lysate was diluted 1:10). The precipitated complexes were washed five times with radioimmune precipitation assay washing buffer (50 mm HEPES-KOH, pH 7.0, 0.5 m LiCl, 1 mm EDTA, 0.7% (w/v) sodium deoxycholate, 1% Igepal CA630) and once with TE buffer (10 mm Tris-HCl, pH 8.0, 1 mm EDTA), and DNA was eluted in 200 μl of elution buffer (lysis buffer without protease inhibitor mixture) after shaking at 65 °C for 6 h. For the ChIP input controls, 100 μl of sonicated cell lysate were diluted 4 times with elution buffer and treated at 65 °C for 6 h. Eluted DNA and input DNA were purified using a PCR purification kit (Qiagen AB, Sollentuna, Sweden) and were then analyzed by a qPCR assay using specific primers for the mouse *Nuak2* intron-enhancer region (forward, 5′-TGAGAAACGACGGAGACAAGCTGCT-3′; reverse, 5′-GTCTGGAGGTTTTGCTGCAGGTCTG-3′), mouse *Pai-1* enhancer (forward, 5′-GTCCAAGAGGAACGAGAACC-3′; reverse, 5′-GGCTTTGTAGGCTCTTGTGG-3′), and mouse *Hbb* (hemoglobin B) gene serving as a control genomic region (forward, 5′-CAACCTGCCCAGGGCCTCAC-3′; reverse, 5′-AGGCTGCTGTCTCTGGCCTGT-3′). The qPCR protocol was as follows: 95 °C for 5 min, followed by 39 cycles of 95 °C for 15 s and 60 °C for 1 min.

### Promoter cloning and luciferase-reporter constructs

The mouse *Nuak2* promoter and enhancer sequences were amplified from genomic DNA isolated from mouse NMuMG cells using primers mapping upstream and downstream of the TSS and upstream and downstream of the intronic enhancer element, which was first identified in a genome-wide screen for SMAD2/3 binding in human epithelial cells (32). For the amplification of the 2-kbp promoter fragment, the primers used were 5′-AGTAGTTGGTGACTGGGTGCAAGGG-3′ (forward) and 5′-GAGTGGGTCGGGCAGCAGTAGCA-3′ (reverse). For the 1-kbp promoter fragment, the primers were 5′-AGTCCTCTTTGATCCTCTGCCAAGTCC-3′ (forward) and 5′-GAGTGGGTCGGGCAGCAGTAGCA-3′ (reverse). For the amplification of the intronic enhancer fragment, the primers used were 5′-GCTCCCCTGACCAACCCCTAAAGAG-3′ (forward) and 5′-CTGGAGCTAGCCGATGGGATGACAA-3′ (reverse). The amplified promoter sequences were cloned into vector pGL4.12 (Promega Corp., Madison, WI) and the enhancer sequence into vector pGL4.24 (Promega Corp., Madison, WI) in one step; the PCR-amplified genomic DNA fragments were blunt-end ligated into the pGL4.12 and pGL4.24 vectors after cutting with EcoRV, producing pGL4.12-mNuak2P-1kbp and pGL4.12-mNuak2P-2kbp (carrying the mouse *Nuak2* promoters only) and pGL4.24-mNuak2/intron (carrying the mouse *Nuak2* enhancer of intron 1). The cloned promoter fragments correspond to 1,102 and 2,274 bp spanning from −1,030 and −2,202 to +63 bp relative to the TSS of the mouse *Nuak2* gene, respectively (Fig. 3*A*). The cloned enhancer fragment corresponds to 420 bp spanning from +2,374 to +2,794 bp relative to the TSS, and the sequence is located in the first intronic region of the *Nuak2* gene (Fig. 3*A*). All *Nuak2* gene bp coordinates are given based on the ENSEMBL NCBIM37 version of the mouse genome. pEGFP-N3 (Takara Bio Europe/Clontech, Saint-Germain-en-Laye, France) was used for normalization of promoter assays.

### Luciferase assays

HEK 293T and NMuMG cells were transiently transfected with the *Nuak2* promoter/enhancer reporter constructs for 36 h prior to stimulation with 5 ng/ml TGFβ for 18 h. pEGFP-N3 (Takara Bio Europe/Clontech) was transfected as control for normalization. The TβRI ALK5 (activin receptor-like kinase 5) mutant pcDNA3-HA-ALK5TD that signals in a constitutive manner has been described previously (7) and was transfected to provide a sustained stimulus of endogenous TGFβ signaling. The pCAGA_12_-MLP-luc reporter and pCMV-β-gal used for normalization were described (43). Transfected cells were lysed in lysis buffer containing 5 mm Tris-phosphate buffer, pH 7.8, 2 mm DTT, 2 mm *trans*-1,2-diaminocyclohexane-*N*,*N*,*N*′,*N*′-tetraacetic acid, 5% glycerol, and 1% Triton X-100. The β-gal assay was performed by mixing the cell lysate with 100 mm sodium phosphate, pH 7.3, 1 mm MgCl_2_, 50 mm β-mercaptoethanol, and 0.67 mg/ml 2-nitrophenyl β-d-galactopyranoside, and the absorbance was monitored at 420 nm. Luciferase reporter assays were performed with the firefly luciferase assay kit from Biotium (Fremont CA) (BTIU30003-2), according to the protocol of the manufacturer. Normalized promoter activity is plotted in bar graphs that represent average values from triplicate determinations with S.D. values.

### Real-time PCR analysis and primers

Total RNA was extracted using RNeasy (Qiagen AB), and cDNA synthesis using a reverse transcription kit (Bio-Rad Laboratories AB) was followed by PCR amplification with the primers indicated in Table 1, as described previously (7, 9).

**Table 1:** **PCR primer sequences used for the quantitative analysis of gene expression** Mouse (mm) and human (hs) primer sequences are listed in the top and bottom, respectively.

Gene	5′ primer sequence	3′ primer sequence
**Mouse**		
mm *Tbp*	CCGCAGTGCCCAGCATCACT	TGGGGAGGCCAAGCCCTGAG
mm *Gadd45*	CTGCATTGCATCCTCATTTCG	GCTCTCCTCGCAGAACAAACTG
mm *Gapdh*	TGTGTCCGTCGTGGATCTGA	CCTGCTTCACCACCTTCTTGA
mm *Smad4*	CATCCTGGACATTACTGGCCA	CCTACCTGAACGTCCATTTCAA
mm *Nuak2*	AGATCGTGTCTGCCCTGCACTA	GCCTTTGTGGTACAGGTTGGAG
mm *Nuak1*	GGTACCTACGGCAAAGTCAAGA	TGAACCATGTCTAGCTCGTCCT
mm *Mark1*	CACGGAGAACCATACGTCTGTG	TGTGAGGCTGTTCATCTGTCG
mm *Mark2*	GCCAAATTTCGCCAGATAGTGT	GTTCATATCAGCATCCAGGAGC
mm *Mark3*	CCTGCTGTGCCCAGTAGTAACA	CTTTGCCGTTCTGAATCACAGA
mm *Mark4*	ACAGCACTAGCACCCCTAACAA	CATTTGGCAACAAGGACGG
mm *Ampk*-α*1*	GTGTGGATTATTGTCACAGGC	TGAAAGACCAAAGTCGGCTATC
mm *Ampk*-α*2*	GAAGATCGGACACTACGTCCTG	TGGCCTGTCAATTGGTGTTC
mm *Sik1*	TTTTACGACGTGGAACGGACC	TGCAACCTGCGTTTTGGTG
mm *Sik2* (*Qik*)	AGCAGATTTCGGCTTTGGAA	AGAACAACTCCCATGCTCCATA
mm *Sik3* (*Qsk*)	TGACAGGTTAATAGCGGAGTGC	CCTTAATGACTGAAGTGTGCGC
mm *Snrk*	AGGCCCAGTTTAGGCAGTCAT	GGCCATTGAGGACATTGTCA
mm *Brsk1*	GCGAGGAGGAAAACCAAGAA	CAGGTCCTGGTCTTCACAGCTA
mm *Brsk2*	GCCACTCCATATGCCATAGAGA	TGCCAAAGTCTGCAATACGG
mm *Melk*	TTAATTTCGTCGTGGCAGTACC	CCACAAGAGAAGGACAGGAGCT
mm *Zeb2*	CACCCAGCTCGAGAGGCATA	CACTCCGTGCACTTGAACTTG
mm *Zeb1*	GCAGGTGAGCAACTGGGAAA	ACAAGACACCGCCGTCATTT
mm *Fn1*	CCCAGACTTATGGTGGCAATTC	AATTTCCGCCTCGAGTCTGA
mm *p15*	CTACCTTTCAGGACGTGGTG	GGCTTTGTGGACGTTGAGTC

**Human**		
hs *HPRT1*	CCCTGGCGTCGTGATTAGT	CACCCTTTCCAAATCCTCAGC
hs *SMAD4*	TGAAGGACTGTTGCAGATAGCA	TCCAGGTGGTAGTGCTGTTATG
hs *GAPDH*	GGAGTCAACGGATTTGGTCGTA	GGCAACAATATCCACTTTACCA
hs *NUAK2*	GATGCACATACGGAGGGAGAT	GCTGGCATACTCCATGACGAT
hs *NUAK1*	GACATGGTTCACATCAGACGA	CAATAGTGCACAGCAGAGACG
hs *MARK1*	ATGTCGGCCCGGACGCCATT	CAGCTTAAGCTCATTTGCTATTTT
hs *MARK2*	TACTTTTCACTGAGGAGGTGGTG	GTGGGCCGACTGGAGAAAG
hs *MARK3*	TTAAAGTCTGAGGACGAGAGCAC	GACTCTCAGGGTAACGGAAGTAG
hs *MARK4*	TGTTACACTGGACTCTAAGCCAC	GTCTGTGTCAGAATCCCTGTCTC
hs *AMPK*-α*1*	TGGTAGGAAAAATCCGCAGAGAA	TTTTCATCCAGCCTTCCATTCTT
hs *AMPK*-α*2*	CGAAGATGGCTGAGAAGCAGAA	GTTCTCCAATCTTCACTTTGCCG
hs *SIK1*	CAGCAGCTATAACCACTTTGCTG	CTGGGCATTCCGATACTCCTTG
hs *SIK2* (*QIK*)	CCTGCTCGTGCTTAAGATTGATG	CAGTCTAAACAAATCAAGCCCCA
hs *SIK3* (*QSK*)	CCCGTATCGGCTACTACGAGAT	ATCTTGATAGCAACCTTGGCCTT
hs *SNRK*	CCTGCCGGCTGAGGAAAAAGA	TTAAATCCTGCCATGCTGGTCC
hs *BRSK1*	CACGACGTCTACGAGAACAAGA	CAGGTAGTCGAATAGCTCACCC
hs *BRSK2*	CCCTACCGGCTGGAGAAGA	CTCACGGTTGACGATCTTGATG
hs *MELK*	AGATTTGATTCCCTTGGCGGG	AGCCACCTGTCCCAATAGTTT
hs fibronectin	CATCGAGCGGATCTGGCCC	GCAGCTGACTCCGTTGCCCA
hs *SERPINE1*	GAGACAGGCAGCTCGGATTC	GGCCTCCCAAAGTGCATTAC
hs *SM22*α	GGTTTATGAAGAAAGCGCAGGAG	CTCTAACTGATGATCTGCCGAGG
hs *TIMP1*	GGGGACACCAGAAGTCAACCAGA	CTTTTCAGAGCCTTGGAGGAGCT
hs calponin	GAGGTTAAGAACAAGCTGGCCC	TTGATGAAGTTGCCGATGTTCTC

### Thymidine incorporation assay

HaCaT and NMuMG cells were seeded in subconfluent conditions in 12-well plates and subsequently were subjected to either *NUAK1 or Nuak2* knockdown, respectively, according to the aforementioned standard siRNA transfection protocol. Twenty-four hours after the second transfection, cells were treated with medium containing 2% FBS in the presence or absence of TGFβ at a final concentration of 5 ng/ml for 24 h, and for the last 6 h, the medium in each well was supplemented with [^3^H]thymidine at a final concentration of 1 μCi/ml. Following wash in ice-cold PBS and fixation with 5% TCA, cells were lysed in 0.1 m NaOH, and the incorporated [^3^H]thymidine was measured by scintillation counting. Every individual experiment was performed in triplicate.

### Living cell analysis of the cell cycle

NMuMG-Fucci cells were transiently transfected with siRNAs twice and stimulated with TGFβ for 24 h as described above. Then fluorescence microscopy and image analysis were performed as described previously (7, 9).

### Collagen gel contraction assay

Three hundred thousand AG1523 cells were seeded in p60 dishes and were transfected according to the standard siRNA transfection protocol described earlier. Following the double transfection, cells were starved overnight with medium containing 0.01% FBS. During the same day, 12-well culture dishes were coated with freshly produced filtered 1% IgG-free BSA in PBS and incubated overnight at 37 °C to block the surface of the dishes, preventing attachment of the newly formed collagen gel. On the next day, AG1523 cells were trypsinized, counted, and seeded into a 1 mg/ml type I collagen solution (PureCol, Advanced BioMatrix Inc., Carlsbad, CA) in ice-cold DMEM at a concentration of 50,000 cells/ml/well. Triplicates were used per condition. After homogenizing the mixture by gentle pipetting, 1 ml of the collagen/cell suspension was added to the BSA-coated dishes, and the solution was incubated for 45 min at 37 °C until the gel was polymerized. Fresh medium containing the indicated treatment conditions was used to supplement the solidified collagen gels, and the contracted surface area was monitored up to 48 h and calculated by employing the ImageJ software.

### Immunofluorescence microscopy

Immunofluorescence microscopy was carried out in NMuMG and AG1523 cells after the indicated siRNA transfection conditions. Following the stated treatments, cells were fixed in 6-well plates for 12 min in 3.7% (w/v) formaldehyde in PBS, followed by permeabilization with 0.1% Triton X-100 in PBS for 10 min, blocked for 60 min with IgG-free 1% BSA in PBS, and incubated overnight at 4 °C with the indicated primary antibodies against αSMA (1:200) (Santa Cruz Biotechnology, sc-32251), fibronectin (1:1,000) (Sigma-Aldrich, F3648), ZO-1 (1:200) (Life Technologies (Stockholm, Sweden), 33-9100) all diluted in 1% BSA/PBS. Following primary antibody incubation, the fixed-permeabilized cells were incubated with anti-mouse or anti-rabbit Alexa Fluor 488– or Alexa Fluor 546–conjugated secondary antibodies (Invitrogen, Thermo Fisher Scientific) at a concentration of 1:1,000 in 1% BSA/PBS for 1 h at room temperature in the dark. Extensive washes were performed between the aforementioned steps. Subsequently, coverslips were set onto glass slides and mounted by using 10 μl of VectaShield HardSet mounting medium containing 4′,6′-diamidino-2-phenylindole (Vector Laboratories, Burlingame, CA) for nuclear visualization. A Zeiss Axioplan 2 fluorescence microscope was used with the Zeiss 40× objective lens. Images were acquired with a Hamamatsu C4742-95 CCD digital camera and the acquisition software QED Camera Plugin version 1.1.6 (QED Imaging Inc., Rockville, MD) and Volocity 1 (PerkinElmer Life Sciences).

### Statistical analysis

Graphs illustrate mean ± S.E. and are based on at least three independent biological experiments, unless stated otherwise. Two-paired Student's *t* test was used to calculate significance; three significance levels are indicated (*, *p* < 0.05; **, *p* < 0.01; ***, *p* < 0.001).

## Author contributions

A. M. and L. P. v. d. H. conceptualization; C. K., E. R., and L. P. v. d. H. validation and generation of new hypotheses; C. K., E. R., M. R., and L. P. v. d. H. investigation; C. K. visualization; C. K., A. M., E. R., M. R., and L. P. v. d. H. methodology; C. K., A. M., and C.-H. H. writing-original draft; A. M., C.-H. H., and P. H. supervision; A. M., C.-H. H., and P. H. funding acquisition; A. M. project administration.

## Supplementary Material

Supporting Information

## Footnotes

6 The abbreviations used are:

TGFβ: transforming growth factor β
TβRI and TβRII: TGFβ type I and II receptor, respectively
WCL: whole-cell lysate(s)
MAPK: mitogen-activated protein kinase
SIK: salt-inducible kinase
AMPK: AMP-activated protein kinase
LKB1: liver kinase B1
mTOR: mammalian target of rapamycin
MLC: myosin regulatory light chain
TSS: transcription start site
EMT: epithelial–mesenchymal transition
αSMA: α-smooth muscle actin
FBS: fetal bovine serum
DMEM: Dulbecco's modified Eagle's medium
qPCR: quantitative PCR
qRT-PCR: quantitative RT-PCR.
