# Supplementary material for: Transforming growth factor β (TGFβ) induces NUAK kinase expression to fine-tune its signaling output
Source: J Biol Chem. 2019 Jan 8;294(11):4119–36. doi: 10.1074/jbc.RA118.004984 (PMC6422081; doi:10.1074/jbc.RA118.004984)
Supplement: Supporting Information [file supp_294_11_4119__index.html]

Transforming growth factor β (TGFβ) induces NUAK kinase expression to fine-tune its signaling output — Opposing roles of TGFβ-induced NUAK1 and NUAK2 — Transforming growth factor β (TGFβ) induces NUAK kinase expression to fine-tune its signaling output — Opposing roles of TGFβ-induced NUAK1 and NUAK2 — Supporting Information 

# Transforming growth factor β (TGFβ) induces NUAK kinase expression to fine-tune its signaling output

## Supporting Information

- Supporting Information (to be published online) - Supporting Figures
