# Supplementary material for: Transforming growth factor β (TGFβ) induces NUAK kinase expression to fine-tune its signaling output
Source: J Biol Chem. 2019 Jan 8;294(11):4119–36. doi: 10.1074/jbc.RA118.004984 (PMC6422081; doi:10.1074/jbc.RA118.004984)
Supplement: Supporting Information [file supp_RA118.004984_139569_1_supp_261570_pkrq0v.pdf]

Transforming growth factor β (TGFβ) induces NUAK kinase expression to fine-tune its signaling output

Constantine Kolliopoulos, Erna Raja, Masoud Razmara, Paraskevi Heldin, Carl-Henrik Heldin, Aristidis Moustakas and Lars P. van der Heide

Supporting Information

This supporting information includes three supporting figures with the corresponding legends.

Supporting Figures

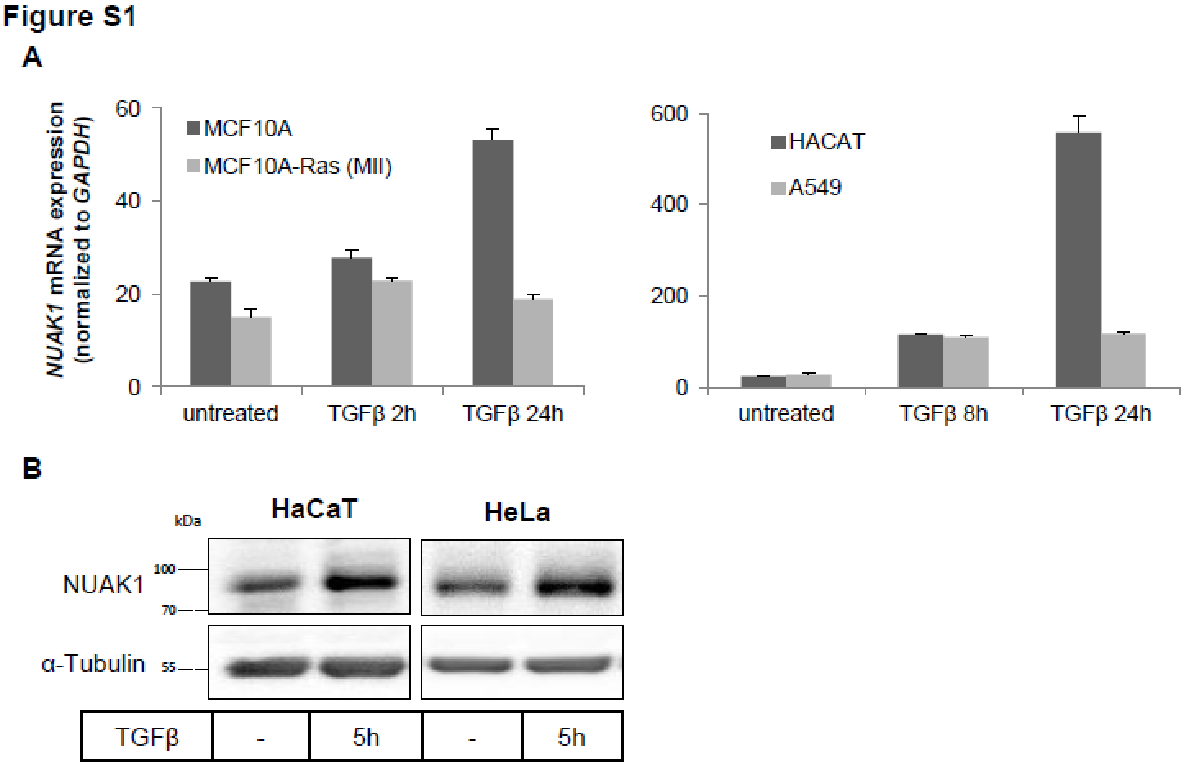

Figure S1. NUAK1 is transcriptionally induced by TGFβ. *A*, Real-time qRT-PCR was performed for *NUAK1* mRNA in a panel of human cell lines (mammary epithelial MCF10A, MCF10ANeoT (MII) cells expressing the oncogenic RAS mutant, lung adenocarcinoma A549 cells and human keratinocytes HaCaT) and normalized to *GAPDH* mRNA at the corresponding time points following TGFβ (5 ng/ml) stimulation. *B*, Immunoblots of NUAK1 and α-tubulin (protein loading control) in low-serum starved (2% FBS, overnight) HaCaT and HeLa cervical carcinoma cells after treatment with fresh 2% FBS/DMEM containing TGFβ (1 ng/ml) for 5 h. Molecular size markers in kDa are shown. All bar graphs show average values derived from triplicate determinations and the corresponding standard deviations. Each independent experiment was repeated at least three times.

Figure S2

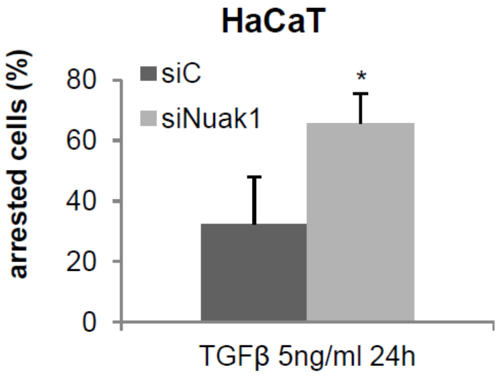

Figure S2. NUAK1 regulates TGFβ-induced epithelial cytostasis. HaCaT cells transfected with scrambled or siRNA targeting *NUAK1*, were starved overnight in 2% FBS/DMEM. Three days after the first transfection, cells were treated with TGFβ (1 ng/ml) for 24 h. During the last 6 h, 3 µCi/ml of [^{3}H]thymidine was added and its incorporation into DNA was measured. Values were normalized to 2% FBS/DMEM treated samples for each siRNA sample. *P*-value was calculated according to Student's paired t-test based on three independent experiments; *P<0.05 was considered statistically significant compared with the scrambled siRNA sample.

Figure S3

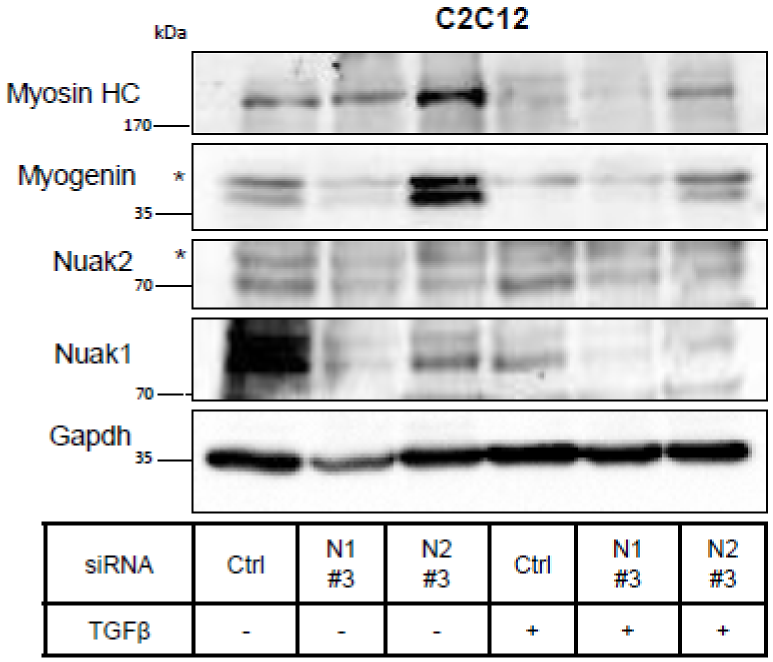

Figure S3. NUAK1 and NUAK2 regulate TGFβ-dependent suppression of myotube differentiation. C2C12 cells transfected with Ctrl or individual and distinct siRNAs targeting *Nuak1* (nr 3) or *Nuak2* (nr 3), were differentiated in 2% horse serum/DMEM in the absence or presence of 5 ng/ml TGFβ and cell extracts were analyzed on day 6 of differentiation, as described in the methods. Representative immunoblot against the indicated proteins demonstrate two differentiation markers (myosin HC and myogenin), the silencing efficiency (Nuak1, Nuak2) and Gapdh as protein loading control. Stars show non-specific protein bands. Molecular size markers in kDa are shown.
